# Sesamol Alleviates Obesity-Related Hepatic Steatosis via Activating Hepatic PKA Pathway

**DOI:** 10.3390/nu12020329

**Published:** 2020-01-26

**Authors:** Hai-Yan Xu, Liang Yu, Ji-Hua Chen, Li-Na Yang, Cui Lin, Xiu-Quan Shi, Hong Qin

**Affiliations:** 1 Department of Nutrition Science and Food Hygiene, Xiangya School of Public Health, Central South University, 110 Xiangya Road, Changsha, Hunan 410078, China; haiyanxu1206@126.com (H.-Y.X.); chenjh@csu.edu.cn (J.-H.C.); ylnly1997@csu.edu.cn (L.-N.Y.); lintto@163.com (C.L.); shixiuquan@126.com (X.-Q.S.); 2 Department of Research and Development Office, Hunan First Normal University, 1015 Fenglinsan Road, Changsha, Hunan 410205, China; kycyuliang@hnfnu.edu.cn

**Keywords:** sesamol, hepatic steatosis, obesity, lipid metabolism, protein kinase A

## Abstract

This study aimed to investigate the effect of sesamol (SEM) on the protein kinase A (PKA) pathway in obesity-related hepatic steatosis treatment by using high-fat diet (HFD)-induced obese mice and a palmitic acid (PA)-treated HepG2 cell line. SEM reduced the body weight gain of obese mice and alleviated related metabolic disorders such as insulin resistance, hyperlipidemia, and systemic inflammation. Furthermore, lipid accumulation in the liver and HepG2 cells was reduced by SEM. SEM downregulated the gene and protein levels of lipogenic regulator factors, and upregulated the gene and protein levels of the regulator factors responsible for lipolysis and fatty acid β-oxidation. Meanwhile, SEM activated AMP-activated protein kinase (AMPK), which might explain the regulatory effect of SEM on fatty acid β-oxidation and lipogenesis. Additionally, the PKA-C and phospho-PKA substrate levels were higher after SEM treatment. Further research found that after pretreatment with the PKA inhibitor, H89, lipid accumulation was increased even with SEM administration in HepG2 cells, and the effect of SEM on lipid metabolism-related regulator factors was abolished by H89. In conclusion, SEM has a positive therapeutic effect on obesity and obesity-related hepatic steatosis by regulating the hepatic lipid metabolism mediated by the PKA pathway.

## 1. Introduction

Obesity is a prevalent metabolic disease worldwide and has become an important public health concern [1]. It is reported that obesity commonly plays a direct role in the onset and progression of non-alcoholic fatty liver disease (NAFLD), which is strongly linked to related metabolic disorders such as insulin resistance, hyperlipidemia, and systemic inflammation [2,3]. NAFLD begins with simple steatosis and then develops to steatohepatitis, before finally progressing to cirrhosis or cancer [4]. Hepatic steatosis is the main pathological feature of NAFLD and the key cause of more severe and progressive disease [5]. Therefore, there is an urgent need to seek an effective strategy to alleviate hepatic steatosis, which will be instrumental in blocking the further progression of NAFLD.

The specific mechanism of obesity-induced hepatic steatosis remains unclear, which makes it difficult to treat NAFLD. Hepatic steatosis is characterized by excessive lipid accumulation in the liver. It is universally known that the liver is the most important metabolic organ responsible for lipid metabolism including lipogenesis, lipolysis, and lipid oxidation [6,7], which maintains lipid homeostasis. Hepatic steatosis occurs when hepatic lipid homeostasis is disrupted by obesity, which disturbs the overall metabolic function of the liver and further exacerbates obesity, forming a vicious cycle [8]. Thus, if there were methods to promote hepatic lipid metabolism by simultaneously regulating the above three metabolic pathways, they would be expected to offer a potential method for hepatic steatosis alleviation.

Recently, there has been increasing interest in phytochemicals due to their beneficial physiological activities for many diseases [9,10,11]. Sesamol (SEM), a kind of natural lignan extracted from sesame oil, possesses multiple biological properties such as anti-inflammatory, anti-dyslipidemia, and anti-oxidation properties [12,13,14,15]. As a multifunctional food component, SEM has aroused considerable interest worldwide. A recent report showed that SEM exerts a preventive role in the onset of obesity [16]. In particular, our previous work suggested that SEM might treat high-fat diet (HFD)-induced obesity and reduce hepatic lipid accumulation [17]. However, the exact molecular mechanisms of SEM responsible for the amelioration of hepatic lipid accumulation have not been fully elucidated. Therefore, the aim of our current study was to identify the therapeutic effect of SEM on obesity-related hepatic steatosis and further investigate the underlying mechanisms through in vivo and in vitro experiments.

## 2. Materials and Methods

### 2.1. Chemicals and Reagents

SEM was purchased from Sigma-Aldrich (St. Louis, MO, USA). Palmitic acid (PA) was purchased from Regent Science (Shenzhen, China). Oil Red O was purchased from Solarbio Science & Technology Co. Ltd. (Beijing, China). Dulbecco’s modified eagle medium (DMEM), penicillin/streptomycin solution, Trizol, radio-immunoprecipitation assay (RIPA) lysis buffer, protease inhibitor, and the thiazolyl blue tetrazolium bromide (MTT) assay kit (GK3605) were purchased from Ding Guo Changsheng Biotechnology Co. Ltd. (Beijing, China). Fetal bovine serum (FBS) was purchased from Tianhang Biotechnology Co. Ltd. (Hangzhou, China). H89 was purchased from MedChemExpress (Monmouth Junction, NJ, USA). The bicinchonininc acid (BCA) protein assay kit (P0010S) was purchased from Beyotime (Shanghai, China). Alanine aminotransferase (ALT, C009-2-1), aspartate aminotransferase (AST, C010-2-1), triacylglycerol (TG, A110-1-1), total cholesterol (TC, A111-1-1), low-density lipoprotein cholesterol (LDL-C, A113-1-1), and high-density lipoprotein cholesterol (HDL-C, A112-1-1) assay kits were obtained from Jiancheng Bioengineering Institute (Nanjing, China). The enzyme-linked immunosorbent assay (ELISA) kits for detecting insulin, β-hydroxybutyrate (β-HB, ml062104), and free fatty acid (FFA, ml002083) was purchased from Enzyme-linked Biotechnology Co. Ltd. (Shanghai, China). The ELISA kits for detecting tumor necrosis factor-α (TNF-α, RK00027) and interleukin-6 (IL-6, RK00008), the primary antibodies against protein kinase A-C (PKA-C, A7715), acetyl CoA carboxylase 1 (ACC1, A15606), phospho-ACC1 (p-ACC1, AP0298), fatty acid synthase (FASN, A0461), peroxisome proliferator-activated receptor-α (PPARα, A11924), peroxisome proliferator-activated receptor gamma coactivator-1α (PGC1α, A12348), carnitine palmitoyltransferase 1α (CPT1α, A5307), AMP-activated protein kinase (AMPK, A12718), phospho-AMPK (p-AMPK, AP0116), histone (A2348), β-actin (AC026), and the secondary antibody horseradish peroxidase (HRP) Goat Anti-Rabbit IgG (AS014) were purchased from Abclonal (Boston, USA). The primary antibodies against hormone-sensitive lipase (HSL, AF6403) and phospho-HSL (p-HSL, AF8026) were purchased from Affinite (Cincinnati, OH, USA), sterol regulatory element binding protein-1c (SREBP-1c, 14088-1-AP) was purchased from Proteintech (Wuhan, China), and phospho-protein kinase A (p-PKA) substrate (9624S) was purchased from Cell Signal Technology (Boston, MA, USA).

### 2.2. Animals and Diet

Eight-week-old male C57BL/6J mice were purchased from Central South University (Hunan, Changsha, China), and kept under standard laboratory conditions (20–24 °C, 40–60% humidity, and 12 h/12 h day/dark cycle). The mice were given one week to acclimate to their new environment and allowed free access to water and food. Then, the mice fed with a normal-fat diet (NFD, 10 kcal% fat, 70 kcal% carbohydrate, 20 kcal% protein; D12450J, Research Diets Inc., USA) formed the control group (NFD group, *n* = 10), and all other mice were fed with a HFD (60 kcal% fat, 20 kcal% carbohydrate, 20 kcal% protein; D12492, Research Diets Inc., USA) for eight weeks to establish the obesity models. Then, the obese mice whose weights were 20% higher than the average weight of the mice in the NFD group were further divided into two groups, including the HFD group (*n* = 10) and the HFD + SEM group (*n* = 10), and all three groups of mice were fed with a HFD for another eight weeks. SEM was dissolved in a vehicle (0.5% carboxylmethylcellulose). Each mouse in the HFD + SEM group was administered SEM by gavage at a dose of 100 mg/kg body weight once daily, and the mice in the NFD and HFD groups were given an equal volume of vehicle by gavage. Their food intake level was recorded every day, and their body weights were measured weekly. All animal experiments were performed in accordance with the protocol (Approval Number: XYGW-2019-038) approved by the Institutional Animal Care and Use Committee of Central South University.

### 2.3. Glucose Tolerance Test (GTT) and Insulin Tolerance Test (ITT)

In the 15^th^ week, the fasting blood glucose (FBG) in the tail vein blood was measured using a glucometer (Contour TS, Bayer, Germany). The mice were intraperitoneally injected with 2 g/kg body weight of glucose after 12 h of fasting for GTT and intraperitoneally injected with an insulin solution at 0.6 U/kg body weight for ITT. Then, the blood glucose levels were monitored with tail blood at 0, 15, 30, 60, and 120 min. The serum insulin levels were determined with an ELISA assay kit. The homeostasis model assessment of insulin resistance (HOMA-IR) was calculated according to the following formula: fasting insulin level (mU/L) × FBG (mmol/L)/22.5.

### 2.4. Serum Parameter Analysis

After 16 weeks, blood samples were collected from the femoral artery and stored overnight at 4 °C. Then, the serum was isolated by centrifuging the samples at 3000× rpm for 15 min. The serum concentrations of triacylglycerol (TG), total cholesterol (TC), low-density lipoprotein cholesterol (LDL-C), high-density lipoprotein cholesterol (HDL-C), alanine aminotransferase (ALT), and aspartate aminotransferase (AST) were determined using commercially available kits. Serum free fatty acid (FFA), β-hydroxybutyrate (β-HB), tumor necrosis factor-α (TNF-α), and interleukin-6 (IL-6) were measured by an ELISA assay.

### 2.5. Histological Analysis

After the mice were killed by cervical dislocation, subcutaneous, epididymal, perirenal white adipose tissues (WATs), and liver were collected, washed with normal saline, and weighed immediately. The WATs and livers were fixed with 4% paraformaldehyde and embedded in paraffin. Five micrometer thick sections were cut and stained with hematoxylin and eosin (H&E). Then, the liver tissues fixed in 4% paraformaldehyde were embedded at an optimum cutting temperature for the frozen sections, and the sections were stained with Oil Red O. All sections were then captured by an optical microscope. Adipocyte size was measured in five fields per sample using ImageJ software.

### 2.6. Hepatic Parameter Analysis

For hepatic lipid content measurement, the liver tissue (200 mg) was homogenized with normal saline (2 mL). The homogenate (400 μL) was mixed with chloroform/methanol (2:1, 4 mL), and then incubated overnight at room temperature. After adding distilled water (800 μL), the mixture was centrifuged at 1000× rpm for 10 min, and the lower lipid phase was collected and lyophilized. The total lipid powder was dissolved in chloroform/methanol (2:1), and liver TG, TC, and FFA were measured by the same kits used for serum analysis.

For the measurement of other parameters, the liver tissue (50 mg) was homogenized with normal saline (450 μL), then the homogenate was centrifuged at 1000× rpm for 10 min at 4 °C. The supernatant was collected to measure liver β-HB, TNF-α, and IL-6 with the same ELISA kits used for serum analysis.

### 2.7. Cell Culture and Treatment

HepG2 cells were purchased from the Peking Union Cell Center (Beijing, China) and cultured in DMEM containing 10% FBS and 1% penicillin/streptomycin solution (100 units/mL penicillin and 100 μg/mL streptomycin). Then, HepG2 cells were maintained at sub-confluent conditions in a humidified incubator with ambient oxygen and 5% CO_2_ at 37 °C. MTT assay kits were used to analyze the effects of PA and SEM on cell proliferation to determine the intervention concentration, followed by the treatment of the cells with or without 0.25 mM PA dissolved in BSA for 24 h, as previously described [18]. Next, the cells exposed to PA were incubated with SEM dissolved in DMSO at different concentrations (12.5, 25, and 50 μM) for 24 h. The control cells were treated with the corresponding concentrations of BSA and DMSO. In the group with the PKA inhibitor, H89 was used to pretreat HepG2 cells at a dose of 20 μM for 1 h prior to SEM treatment.

### 2.8. Cellular Oil Red O Staining and Lipid Content

HepG2 cells were fixed with 4% paraformaldehyde for 15 min and then washed with 60% isopropanol. Oil Red O solution was used to stain intracellular lipid droplets at room temperature for 1 h. After that, the cells were washed three times with PBS and photographed under a microscope. Intracellular Oil Red O was solubilized with 100% isopropanol and was assessed by an absorbance measurement at 510 nm using a microplate reader for quantitative analysis. Intracellular TG, TC, LDL-C, and HDL-C were measured by the same kits used for the serum analysis.

### 2.9. Quantitative Reverse Transcription-Polymerase Chain Reaction (RT-PCR)

Total RNA was isolated from the livers or HepG2 cells using a Trizol reagent and was reverse transcripted using the HiScriptIIQ RT SuperMix (Vazyme, Nanjing, China), as per the manufacturer’s instructions. Quantitative RT-PCR reactions using ChamQ Universal SYBR qPCR Master Mix (Vazyme, Nanjing, China) were run on a RT-PCR Detection System (LightCycler 480 II, Roche, Basel, Switzerland). Ct values were normalized to β-actin, and the relative gene expressions were calculated with the 2^-△△Ct^ algorithm. The primer sequences used in this study are shown in Table 1.

### 2.10. Western Blot

Liver tissue (20 mg) was homogenized in 400 μL RIPA lysis buffer with a protease inhibitor; HepG2 cells were lysed in 150 μL RIPA lysis buffer with a protease inhibitor. All homogenates or lysates were then centrifuged at 14000× rpm for 15 min at 4 °C, and protein supernatants were transferred to new centrifugal tubes. The protein level was determined by BCA assay kits for the western blot analysis. The protein samples (35 µg) were separated by SDS-PAGE and then transferred to polyvinylidene fluoride (PVDF) membranes, as previously described [19]. The membranes were blotted overnight at 4 °C using appropriate primary antibodies and then incubated with the secondary antibody for 1 h at room temperature. Immunoreactive bands were visualized by electrochemiluminescence (ECL) and detected using the chemiluminescence imager (Tanon-5500, Shanghai, China). Band densities were quantified by Tanon Gis software.

### 2.11. Statistical Analysis

All data were presented as the mean ± standard deviation (SD), and statistical analysis was performed using SPSS 20.0 (Chicago, IL, USA). One-way analysis of variance (ANOVA) was used for multiple comparisons, and Fischer least-significant difference (LSD) test was used to compare the results between two groups if it was statistically significant on ANOVA. *p* < 0.05 was considered statistically significant.

## 3. Results

### 3.1. Sesamol (SEM) Ameliorated High-Fat Diet (HFD)-Induced Obesity

**Figure 1:**
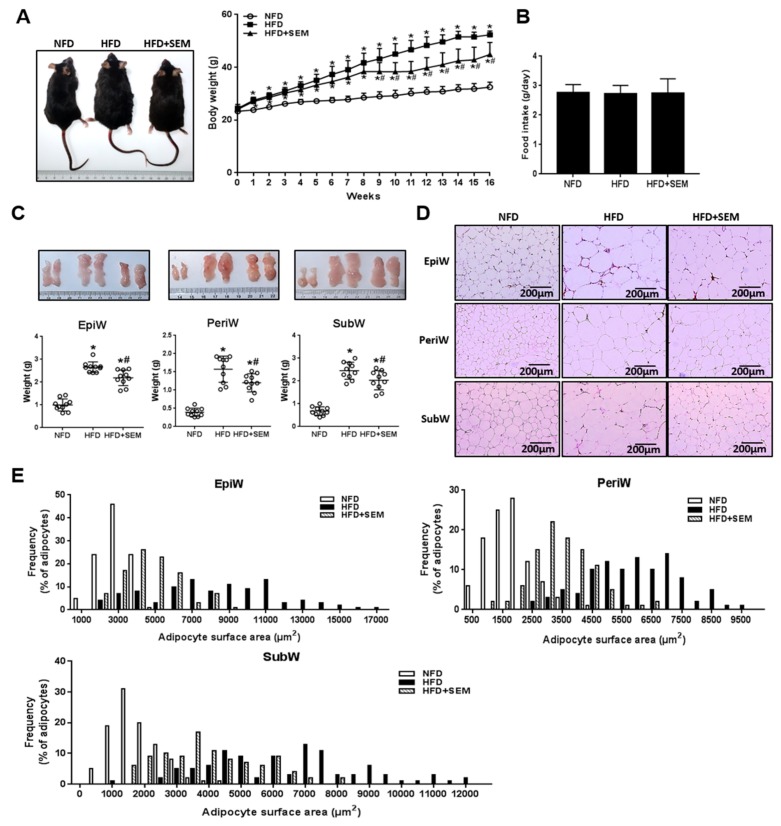
**Sesamol** (SEM) reduces body weight and fat mass in high-fat diet (HFD)-induced obese mice. (**A**) Representational shape view and body weight curve. (**B**) Food intake. (**C**) Representative morphology photos and weights of EpiW, PeriW, and SubW. (**D**) Hematoxylin and eosin (H&E) staining (200×), scale bar = 200 μm. (**E**) Size distribution of adipocytes. EpiW: epididymal WAT, PeriW: perirenal WAT, SubW: subcutaneous WAT. All values are presented as mean ± SD (*n* = 10). * *p* < 0.05 versus the normal-fat diet (NFD) group; ^#^ *p* < 0.05 versus the HFD group.

To examine the effect of SEM on the development of obesity, HFD-induced obese mice were administered SEM by gavage for eight weeks. During the experimental period, we measured the body weights of the mice once a week and found that SEM significantly decreased the body weight gain of the obese mice (Figure 1A), while the food intake was not affected by SEM (Figure 1B). We next sought to determine whether the decrease in body weight was associated with a decrease in fat mass. As expected, SEM significantly reduced the weights of the epididymal, perirenal, and subcutaneous WATs (Figure 1C). Moreover, SEM-treated mice had a smaller adipocytes size than those in the HFD group (Figure 1D,E). Taken together, these data indicate that SEM administration ameliorated HFD-induced obesity and decreased the fat mass in obese mice.

### 3.2. SEM Attenuated Metabolic Disorders in Obese Mice

**Figure 2:**
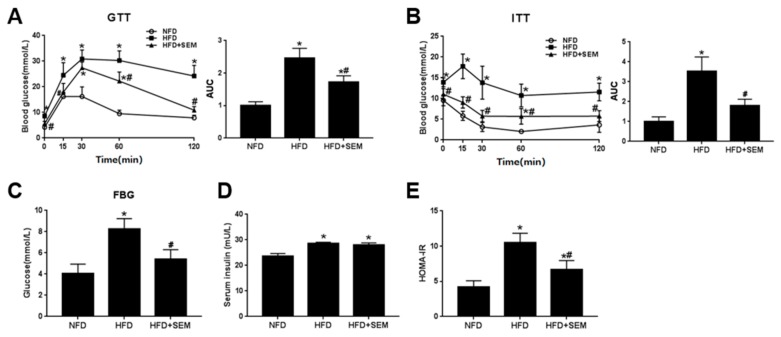
SEM improves insulin sensitivity in HFD-induced obese mice. (**A**) Glucose tolerance test (GTT) curve and area under the curve (AUC). (**B**) Insulin tolerance test (ITT) curve and AUC. (**C**) Fasting blood glucose levels. (**D**) Serum insulin levels. (**E**) Homeostasis model assessment of insulin resistance (HOMA-IR) levels. All values are presented as mean ± SD (*n* = 5). * *p* < 0.05 versus the NFD group; ^#^ *p* < 0.05 versus the HFD group.

To detect the effects of SEM on obesity-related metabolic disorders, we first examined the changes of insulin sensitivity in the obese mice. The results showed that glucose disposal was delayed during the GTT and ITT of the obese mice in the HFD group, and SEM markedly promoted glucose disposal (Figure 2A,B), which indicated that glucose and insulin tolerance was improved after SEM treatment. The level of FBG was also significantly decreased by SEM (Figure 2C). Moreover, although there was no difference in the serum insulin level (Figure 2D), the HOMA-IR was lower in the HFD + SEM group than in the HFD group (Figure E). These data demonstrate that SEM attenuated obesity-related insulin resistance. We next investigated the effects of SEM on the serum lipid profiles in mice. We found that serum TG, TC, LDL-C, and FFA levels were significantly reduced, while the HDL-C level was increased by SEM compared with the HFD group (Table 2), which demonstrated that SEM could ameliorate obesity-related dyslipidemia. The level of serum β-HB, a fatty acid metabolite [20], was increased by SEM (Table 2), which suggested that SEM might promote fatty acid β-oxidation to reduce serum FFA. Due to the systemic low-grade chronic inflammation caused by obesity [21], the serum inflammation cytokine levels were measured. As expected, HFD-induced obese mice had higher TNF-α and IL-6 levels, while the levels of these two indicators were lower in the HFD + SEM group than in the HFD group (Table 2). Thus, we concluded that SEM could attenuate obesity-related metabolic disorders in obese mice.

### 3.3. SEM Alleviated Hepatic Steatosis in Obese Mice

**Figure 3:**
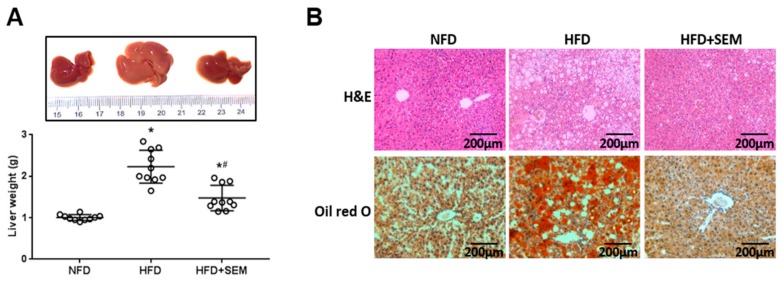
Effects of SEM on hepatic lipid accumulation. (**A**) Representative morphology photos and weights of livers. (**B**) H&E staining (200×) and Oil Red O staining (200×), scale bar = 200 μm. All values are presented as mean ± SD (*n* = 10). * *p* < 0.05 versus the NFD group; ^#^ *p* < 0.05 versus the HFD group.

We sought to further determine the role of SEM in the liver. Obesity-related hepatic steatosis results from abnormal lipid accumulation in the liver, and eventually leads to liver injury [22]. We found that the appearance of the livers in the HFD group was pale and large, which was modified after SEM treatment. The liver weights of the SEM-treated mice were lower compared to the HFD group (Figure 3A). The results from the H&E and Oil Red O staining showed that the fat vacuoles and lipid droplets accumulated significantly in the livers of mice fed HFD and were clearly reversed by SEM treatment (Figure 3B). Consistent with these observations, the levels of liver TG and FFA decreased. In addition, liver β-HB was increased by SEM, although there was no significant difference in the liver TC level between the HFD group and the HFD + SEM group (Table 3), indicating that SEM might modify hepatic lipid profiles and enhance hepatic fatty acid β-oxidation. Furthermore, compared with the HFD group, SEM treatment decreased the levels of serum ALT, AST, liver TNF-α, and IL-6 (Table 3), which suggests that SEM reduced liver injury and inflammation. These findings show that SEM could attenuate hepatic steatosis and improve liver function in obese mice.

### 3.4. SEM Promoted Hepatic Lipid Metabolism in Obese Mice

**Figure 4:**
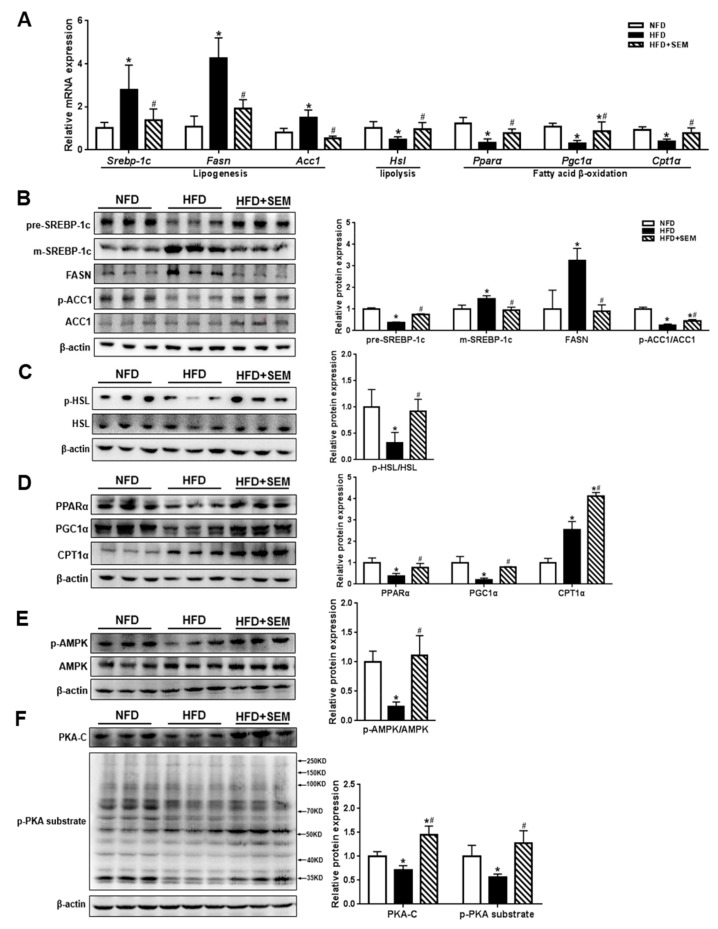
Effects of SEM on regulatory factors involved in hepatic de novo lipogenesis, lipolysis, and fatty acid β-oxidation. (**A**) Gene levels of lipid metabolism regulatory factors. (**B**) Protein levels responsible for de novo lipogenesis and densitometric determinations. (**C**) Protein levels involved in lipolysis and densitometric determinations. (**D**) Protein levels involved in fatty acid β-oxidation and densitometric determinations. (**E**) AMPK and p-AMPK levels and densitometric determinations. (**F**) PKA-C and p-PKA substrate levels for PKA activity and densitometric determinations. All values are presented as mean ± SD (*n* = 3). β-actin was protein loading control.* *p* < 0.05 versus the NFD group; ^#^ *p* < 0.05 versus the HFD group.

To investigate the molecular mechanism of SEM in alleviating hepatic steatosis, we detected the expression of lipid metabolism-related regulatory factors at the gene and protein levels in the liver. Our results showed that the mRNA levels of lipogenic genes including *Srebp-1c* and its target genes such as *Fasn* and *Acc1* were significantly downregulated. In addition, the *Hsl* levels for lipolysis and the *Pparα*, *Pgc1α*, and *Cpt1α* levels for fatty acid β-oxidation were markedly upregulated by SEM (Figure 4A). Correspondingly, the protein expression of the precursor form of SREBP-1c (pre-SREBP-1c) was increased, while the mature form of SREBP-1c (m-SREBP-1c) and FASN levels were decreased by SEM treatment (Figure 4B). In addition, the p-ACC1/ACC1 ratio was increased by SEM treatment, indicating a decrease in ACC1 activity (Figure 4B). The p-HSL/HSL ratio was markedly higher in the HFD + SEM group than in the HFD group (Figure 4C). Furthermore, the PPARα, PGC1α, and CPT1α levels increased after SEM treatment (Figure 4D). Interestingly, the results showed that SEM activated the AMPK pathway by increasing the p-AMPK/AMPK ratio (Figure 4E), which might regulate lipogenesis and fatty acid β-oxidation in the liver of HFD-induced obese mice [23]. Since the phosphorylation activation of HSL depends on the PKA pathway [24], we measured the expression of PKA-C and p-PKA substrates for PKA activity, and the results showed that SEM significantly increased the PKA-C and p-PKA substrate levels (Figure 4F). These findings indicated that SEM markedly promoted hepatic lipid metabolism by suppressing de novo lipogenesis, enhancing lipolysis and fatty acid β-oxidation in the livers of obese mice.

### 3.5. SEM Reduced Lipid Accumulation in Palmitic Acid (PA)-Treated HepG2 Cells

**Figure 5:**
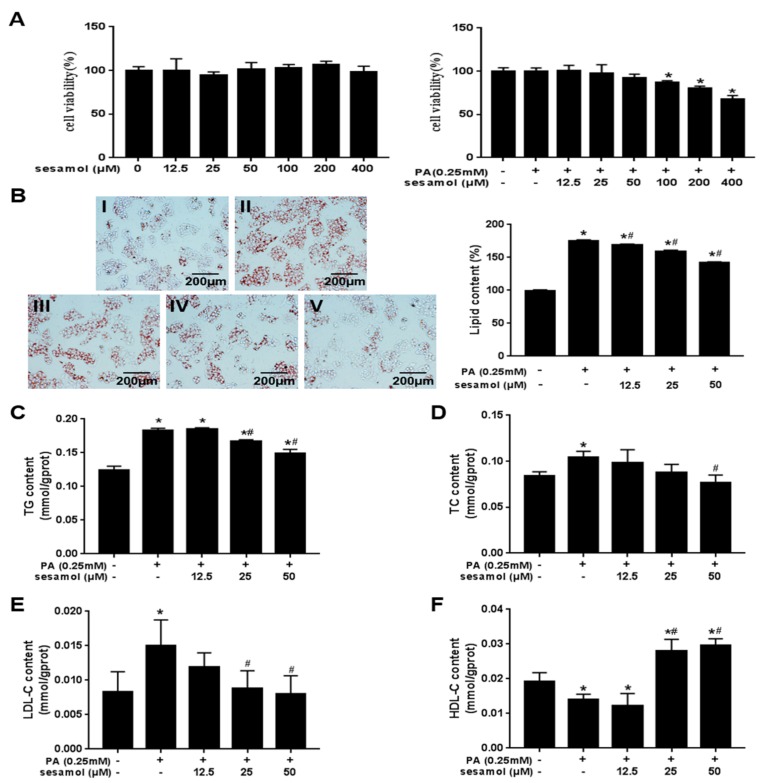
Effects of SEM on cell viability and intracellular lipid accumulation. (**A**) MTT assay for cell viability. (**B**) Oil Red O staining and intracellular lipid content measured by semi quantitative. Images were obtained at ×200. Scale bars = 200 μm. I: Bovine serum albumin + dimethyl sulfoxide (BSA + DMSO); II: Palmitic acid (PA) + DMSO; III: PA + SEM (12.5 μM); IV: PA + SEM (25 μM); V: PA + SEM (50 μM). (**C**) Triacylglycerol (TG) level, (**D**) total cholesterol (TC) level, (**E**) low-density lipoprotein cholesterol (LDL-C) level, and (**F**) high-density lipoprotein cholesterol (HDL-C) level in HepG2 cells. All values are presented as mean ± SD (*n* = 3). * *p <* 0.05 versus the BSA + DMSO group; ^#^ *p <* 0.05 versus the PA + DMSO group.

Three concentrations of SEM (12.5, 25, and 50 μM) were used in this experiment, according to the results of the MTT assay (Figure 5A). PA is known to induce intracellular lipid accumulation [25], and we observed that SEM dose-dependently reduced intracellular lipid droplets (Figure 5B). Furthermore, SEM also markedly reduced intracellular TG, TC, and LDL-C levels (Figure 5C–E) and increased HDL-C levels (Figure 5F). These results indicated that SEM decreased the lipid accumulation of HepG2 cells in the presence of high PA concentration.

### 3.6. SEM Promoted Lipid Metabolism in PA-Treated HepG2 Cells

**Figure 6:**
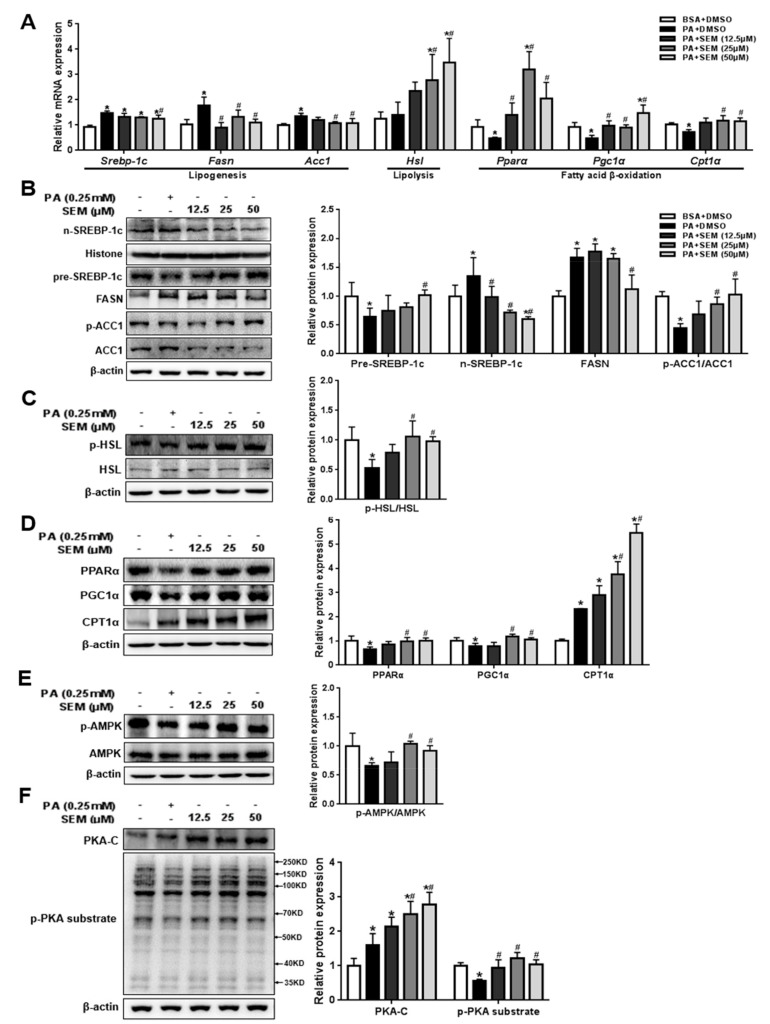
Effects of SEM on regulatory factors involved in de novo lipogenesis, lipolysis, and fatty acid β-oxidation in PA-treated HepG2 cells. (**A**) Gene levels of regulatory factors. (**B**) Protein levels responsible for de novo lipogenesis and densitometric determinations. (**C**) Protein levels involved in lipolysis and densitometric determinations. (**D**) Protein levels involved in fatty acid β-oxidation and densitometric determinations. (**E**) AMPK and p-AMPK levels and densitometric determinations. (**F**) PKA-C and p-PKA substrate levels and densitometric determinations. All values are presented as mean ± SD (*n* = 3). β-actin and histone were protein loading control. * *p <* 0.05 versus the BSA + DMSO group; ^#^ *p <* 0.05 versus the PA + DMSO group.

We next investigated the molecular mechanism by which SEM reduces intracellular lipid accumulation. At the genetic level, SEM significantly reduced the mRNA levels of lipogenic genes such as *Srebp-1c*, *Fasn,* and *Acc1*. Furthermore, SEM markedly increased the *Hsl* level for lipolysis and the *Pparα*, *Pgc1α*, and *Cpt1α* levels for fatty acid β-oxidation (Figure 6A). At the protein level, we found that SEM dose-dependently increased the level of the precursor form of SREBP-1c in the cytoplasm and reduced the mature form of SREBP-1c in the nucleus (Figure 6B). Likewise, the FASN level was reduced, and the p-ACC1/ACC1 ratio was increased by SEM in a dose-dependent manner (Figure 6B). We further discovered that SEM significantly increased the p-HSL/HSL ratio (Figure 6C). In addition, the PPARα, PGC1α, and CPT1α levels were also increased by SEM (Figure 6D). Similar to the results in vivo, we found that SEM markedly increased the p-AMPK/AMPK ratio (Figure 6E). SEM also increased the levels of the PKA-C and p-PKA substrates in a dose-dependent manner (Figure 6F). Taken together, SEM might suppress intracellular de novo lipogenesis and promote fatty acid β-oxidation by activating AMPK as well as enhance lipolysis through the PKA-dependent pathway, resulting in the promotion of lipid metabolism in HepG2 cells.

### 3.7. SEM Regulated Lipid Homeostasis by Activating the Hepatic Protein Kinase A (PKA) Pathway

**Figure 7:**
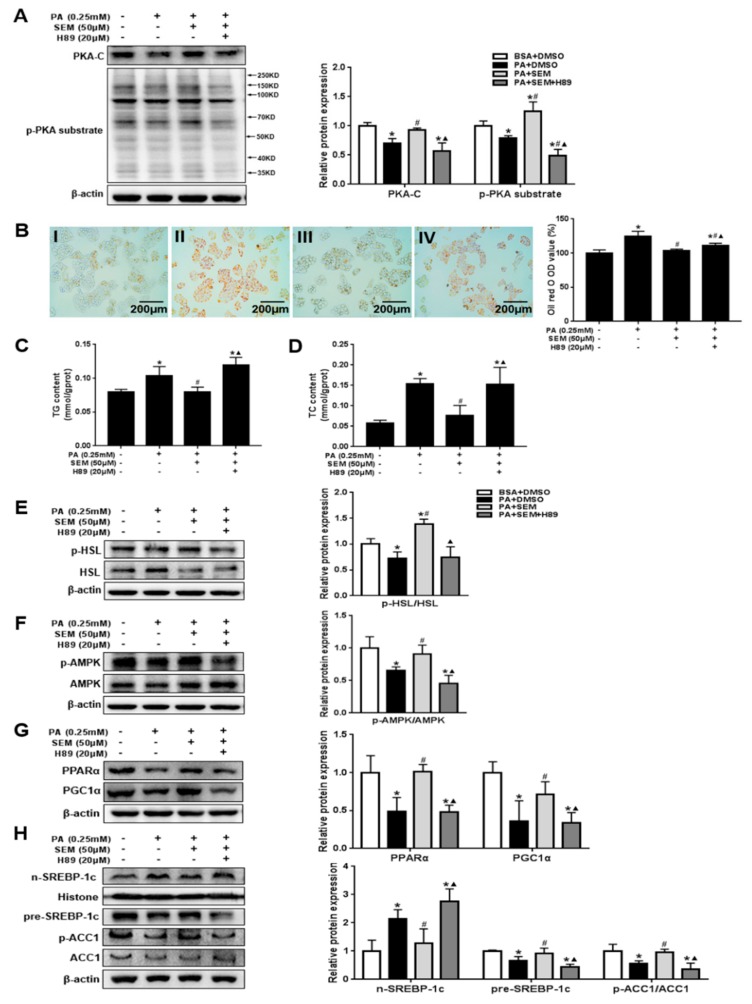
PKA mediates the effects of SEM on lipid metabolism in PA-treated HepG2 cells. (**A**) PKA-C, p-PKA substrate levels, and densitometric determinations. (**B**) Oil Red O staining and intracellular lipid content measured by semi quantitative. Images were obtained at ×200. Scale bars = 200 μm. I: BSA + DMSO; II: PA + DMSO; III: PA + SEM; IV: PA + SEM + H89. (**C**) TG level. (**D**) TC level. (**E**) p-HSL, HSL levels, and densitometric determinations. (**F**) p-AMPK, AMPK levels, and densitometric determinations. (**G**) Protein levels responsible for fatty acid β-oxidation and densitometric determinations. (**H**) Protein levels responsible for de novo lipogenesis and densitometric determinations. All values are presented as mean ± SD (*n* = 3). β-actin and histone were protein loading control. * *p* < 0.05 versus the BSA + DMSO group; ^#^ *p* < 0.05 versus the PA + DMSO group; ^▲^ *p* < 0.05 versus the PA + SEM group.

To further establish PKA’s role in mediating the effects of SEM, we pretreated HepG2 cells with H89 to suppress PKA activity and then treated the cells with SEM. Certainly, the SEM-induced increases in the PKA-C and p-PKA substrate levels were abolished by pretreatment with H89 (Figure 7A). We observed that SEM caused no reduction of lipid droplets in the presence of H89 (Figure 7B). Consistently, SEM failed to decrease the TG and TC levels in PA-treated HepG2 cells when PKA activity was inhibited by H89 (Figure 7C,D). These results further demonstrated that the inhibition of PKA activity blocked the effects of SEM on the p-HSL/HSL and p-AMPK/AMPK ratio (Figure 7E,F). Furthermore, the downstream protein levels of AMPK responsible for lipogenesis and fatty acid β-oxidation were reversed with H89 (Figure 7G,H). Collectively, apart from activating PKA-dependent lipolysis, SEM could activate the PKA/AMPK pathway to regulate the lipid homeostasis of HepG2 cells in the presence of high PA concentration.

## 4. Discussion

In the current study, we explored the novel therapeutic effects and molecular mechanisms of SEM on obesity-related hepatic steatosis by using HFD-induced obese C57BL/6J mice models and PA-treated HepG2 cells. Our findings showed that SEM intervention reduced the body weight gain of obese mice and alleviated obesity-related metabolic disorders including insulin resistance, hyperlipidemia, and systemic inflammation. More importantly, SEM significantly decreased hepatic fat deposition and improved liver functions. At the molecular level, we first demonstrated that SEM activated the PKA/AMPK signal to decrease de novo lipogenesis and increase fatty acid β-oxidation as well as stimulated the PKA-dependent pathway to enhance lipolysis in the liver. Taken together, SEM has a positive therapeutic effect on hepatic steatosis, which might be due to the fact that sesamol promotes hepatic lipid metabolism by activating the PKA pathway.

Hepatic steatosis is common in obese individuals and is the initial pathological feature of NAFLD that is closely associated with multiple metabolic disorders such as insulin resistance, hyperlipidemia, and systemic inflammation [2,3,26]. In the current study, HFD-fed mice had a higher body weight, and, after SEM treatment, we found that this body weight decreased without affecting food intake, and the fat mass was obviously reduced. Moreover, our results showed that the glucose disposal was improved in GTT and ITT, moreover, FBG and HOMA-IR were markedly decreased, which strongly indicated that sesamol decreased insulin resistance rather than the insulin levels themselves. SEM also modified the serum lipid profiles and reduced the serum inflammatory factor levels in obese mice. The findings indicate the positive effects of SEM on obesity and related metabolic disorders. These beneficial effects are strongly linked to a decrease in lipid deposition in the body [27]. Since the liver is the main metabolic organ involved in lipid metabolism [28], we speculate that these changes might indicate the promotion of hepatic lipid metabolism to increase extra lipid consumption. The liver is known to be prone to accumulate fat, especially under the condition of obesity [29], and we found that SEM markedly reduced lipid droplet deposition and modified lipid profiles in the liver. In addition, SEM intervention decreased hepatic injury and inflammatory levels. The HepG2 cell line has been widely used to explore hepatic metabolism in vitro due to its similar metabolic properties to normal human hepatocytes [30,31]. In this study, we used HepG2 cells treated with PA to mimic a high-fat environment in vivo. Similarly, SEM also reduced intracellular lipid accumulation. These results indicate that SEM might have a therapeutic effect on hepatic steatosis and improve liver function, which is also consistent with our hypothesis that SEM could enhance hepatic lipid metabolism to alleviate obesity and metabolic disorders.

The ectopic lipid accumulation in hepatocytes is primarily determined by lipogenesis and lipid catabolism (lipolysis and fatty acid oxidation) [32,33]. Since SEM might reduce lipid accumulation in the liver, we next sought to explore whether SEM could regulate hepatic lipid metabolism. One of the processes of hepatic lipid metabolism is lipogenesis. SREBP-1c is a membrane-bound transcription factor that controls hepatic de novo expression of lipogenic enzymes such as FASN and ACC1 [34]. Notably, SREBP-1c has transcriptional activity when it is processed into a mature form and transported into the nucleus [35]. In our study, we proved that SEM significantly reduced the levels of lipogenic genes such as *Srebp-1c*, *Fasn*, and *Acc1*. Correspondingly, the protein levels of m-SREBP-1c and FASN were decreased, while the pre-SREBP-1c level was increased by SEM. ACC1 activity can be suppressed when its serine 79 site is phosphorylated [36], and our results demonstrate that the ratio of p-ACC1/ACC1 is relatively higher with SEM treatment. These results suggest that SEM might inhibit hepatic lipogenesis in hepatic steatosis.

Another process of hepatic lipid metabolism is lipid catabolism. The decrease of hepatic lipid droplets is probably due to an increase in lipolysis. HSL is a critical rate-limiting enzyme for lipolysis. It was reported that lipolysis mediated by HSL can reduce hepatic steatosis [37]. HSL can be activated by phosphorylation at the serine 660 site; then, p-HSL translocates from the cytoplasm to lipid droplets to catalyze TG hydrolysis [38]. Our findings demonstrate that SEM increases the gene level of *Hsl* and the p-HSL/HSL ratio, thereby indicating the enhancement of hepatic lipolysis. However, an increase in lipolysis might lead to excessive FFA releasing into blood [39], which could cause insulin resistance and deteriorate NAFLD [40,41]. In our work, even when the lipolysis in the liver was increased, serum and liver FFA levels were both reduced. In addition, insulin resistance was alleviated by SEM. We thus hypothesize that hepatic fatty acid β-oxidation might also be increased by SEM. In this study, the level of β-HB, which is a metabolite of fatty acid β-oxidation [20], was increased in the serum and liver by SEM, which further confirms the feasibility of our conjecture. Fatty acid β-oxidation is regulated by some key transcription factors such as PPARα [42]. PPARα is highly expressed in the liver and promotes fatty acid β-oxidation by stimulating the transcription of the related rate-limiting enzyme, CPT1α, which further increases ketone body (β-HB) synthesis [43]. PGC1α, a key transcription cofactor for activating PPARα, is involved in mitochondrial biosynthesis [44], so it also plays a vital role in fatty acid β-oxidation. Here, we showed that the gene levels of *Pparα*, *Pgc1α*, and *Cpt1α* were markedly upregulated by SEM. Additionally, the hepatic protein expression of PPARα, PGC1α, and CPT1α was accordingly increased. These findings strongly demonstrate that SEM promotes hepatic lipolysis and fatty acid β-oxidation in hepatic steatosis.

AMPK, a universal metabolic sensor, responds to the intracellular energy state and plays a key role in the maintenance of lipid homeostasis [45]. It is well documented that AMPK is activated by phosphorylation at Thr172 and positively regulated fatty acid β-oxidation by activating PPARα and PGC-1α [46,47]. Therefore, we measured the regulation of AMPK by SEM. As expected, the results showed that the p-AMPK/AMPK ratio was obviously higher with SEM intervention, whether in vivo or in vitro. Furthermore, in the liver, activation of AMPK suppresses the maturation of SREBP-1c and increases p-ACC1 (Ser 79) to reduce lipogenesis, while the inactivation of ACC1 results in an increase of CPT1α [48,49], which is further responsible for the effects of SEM in repressing lipogenesis and promoting fatty acid β-oxidation. Likewise, a recent study found that SEM might reduce fat accumulation by activating the AMPK signal in 3T3-L1 adipocytes [50]. Collectively, we conclude that SEM reduces hepatic lipogenesis and increases fatty acid β-oxidation via the AMPK pathway.

Several studies have shown that the AMPK pathway might be activated by PKA to regulate lipogenesis and fatty acid β-oxidation [51]. It is also known that PKA, a typical upstream of HSL, is closely associated with lipolysis [38]. Thus, we assumed that PKA is a key factor to maintain hepatic lipid homeostasis by regulating these three pathways of lipid metabolism. PKA is composed of two regulatory (R) subunits and two catalytic (C) subunits. Furthermore, cAMP can bind to the R subunit and release an active monomer C subunit to activate PKA [52]. Active PKA-C may phosphorylate downstream substrates including HSL and AMPK to regulate lipid metabolism [53]. Our results demonstrate that hepatic PKA-C and p-PKA substrate levels were increased by SEM treatment, which demonstrates that SEM can activate the PKA signal. To further confirm that the therapeutic effect of SEM on hepatic steatosis was due to the activation of PKA in the liver, we examined the lipid accumulation and regulation of PKA downstream signals in PA-treated HepG2 cells in the presence of the PKA inhibitor, H89. We found that the effect of SEM in decreasing lipid accumulation was abolished. In addition, the SEM-induced downregulation of lipogenic factors and the upregulation of lipolysis and fatty acid β-oxidation-related factors were completely reversed along with the inhibition of PKA activity. These data demonstrate that SEM can not only stimulate the PKA-dependent lipolysis, but can also activate the PKA/AMPK pathway to reduce lipogenesis and increase fatty acid β-oxidation, which is instrumental in improving hepatic lipid metabolism. Our results thus offer new insights into the potential mechanism responsible for the hepatoprotective bioactivities of SEM. However, additional experiments should be conducted in the future with more persuasive animal models such as liver-specific PKA-overexpression or knockout mice to determine a more explicit regulatory mechanism for SEM. Furthermore, there is a lack of evidence for SEM’s role in alleviating human hepatic steatosis or NAFLD. Therefore, long-term prospective clinical studies are required to further assess the effects of SEM.

## 5. Conclusions

In summary, this study provides new evidence that SEM might alleviate obesity-related hepatic steatosis via the PKA pathway. Activation of the PKA signal promoted hepatic lipid metabolism by suppressing lipogenesis, promoting lipolysis, and fatty acid β-oxidation, which was the core mechanisms of SEM to reduce obesity-related hepatic excessive fat accumulation. Based on the positive effect of SEM on hepatic steatosis and obesity in the current study, SEM could be developed as a novel therapy targeting PKA to treat NAFLD, obesity, and obesity-related systemic metabolic disorders in the future.

## Figures and Tables

**Table 1:** The primer sequences used for quantitative reverse transcription-polymerase chain reaction (RT-PCR).

Specifies	Gene Name	Primer Sequence (5′ to 3′)	
		Forward	Reverse
**Mus musculus**	*Srebp-1c*	AGGCAGAGAGCAGAGATG	AAAGAGAAGAGCCAAGCA
	*Acc1*	AAAACAGGGAGGAAGCAA	TCACCCCGAATAGACAGC
	*Fasn*	ACCTCATTGGTGGTGTGG	CATTGTGTGTGCCTGCTT
	*Hsl*	CCGCTATGTGGCTTCTAA	CACTCCTGGTCGGTTGAT
	*Cpt1α*	CTATTCGTCTTCTGGGAT	GTGTTGGATGGTGTCTGT
	*Pparα*	TCTCCCCACATCCTTTCT	CTGCCGTTGTCTGTCACT
	*Pgc1α*	GCCTTCTTGCTCTTCCTT	ATCCTTTGGGGTCTTTGA
	*β-actin*	CGTGCGTGACATCAAAGA	AAGGAAGGCTGGAAAAGA
Homo sapiens	*Srebp-1c*	GCAACACAGCAACCAGAA	GAAAGGTGAGCCAGCATC
	*Acc1*	AAGACTGGGTAGAGCGAT	GGGAAACTGACAGAGGAC
	*Fasn*	GCCCAAGGGAAGCACATT	CGAAGCCACCCAGACCAC
	*Hsl*	TGGAGGAGTGCTTCTTCG	GATTCGTTCCCCTGTTGA
	*Cpt1α*	CTACTTCCAGACTTGCCC	ACACCATTTCCATTCCAC
	*Pparα*	TAGGGACAGACTGACACC	CATAACAAAAGATACGGG
	*Pgc1α*	TGCCACCACCATCAAAGA	ACCAAACAGCCGCAGACT
	*β-actin*	TTGCGTTACACCCTTTCT	ACCTTCACCGTTCCAGTT

**Table 2:** Effects of sesamol (SEM) on serum metabolic parameters.

	NFD	HFD	HFD+SEM
Serum TG (mM)	0.74 ± 0.10	1.59 ± 0.42 *	0.93 ± 0.17 ^#^
Serum TC (mM)	5.90 ± 0.96	13.38 ± 3.03 *	10.03 ± 0.93 ^#^
Serum LDL-C (mM)	0.16 ± 0.03	0.24 ± 0.03 *	0.18 ± 0.03 ^#^
Serum HDL-C (mM)	2.43 ± 0.55	2.53 ± 0.15	3.79 ± 0.15 *^#^
Serum FFA (μM)	66.69 ± 2.82	76.07 ± 3.40 *	63.07 ± 2.62 ^#^
Serum β-HB (mM)	0.14 ± 0.02	0.11 ± 0.01 *	0.13 ± 0.01 ^#^
Serum TNF-α (pg/mL)	11.79 ± 0.78	13.92 ± 1.72 *	11.17 ± 0.73 ^#^
Serum IL-6 (pg/mL)	11.07 ± 0.61	14.8 ± 1.98 *	12.07 ± 1.12 ^#^

All values are presented as mean ± SD (*n* = 5). * *p* < 0.05 versus the NFD group; ^#^ *p* < 0.05 versus the HFD group. NFD: normal-fat diet group, HFD: high-fat diet group, HFD+SEM: HFD+SEM group, TG: triacylglycerol, TC: total cholesterol, LDL-C: low-density lipoprotein cholesterol, HDL-C: high-density lipoprotein cholesterol, ALT: alanine aminotransferase, AST: aspartate aminotransferase, FFA: free fatty acid, β-HB: β-hydroxybutyrate, TNF-α: tumor necrosis factor-α, IL-6: interleukin-6.

**Table 3:** Effects of SEM on hepatic lipid profiles and liver function.

	NFD	HFD	HFD+SEM
Liver TG (mmol/gprot)	0.16 ± 0.03	0.28 ± 0.09 *	0.13 ± 0.03 ^#^
Liver TC (mmol/gprot)	0.05 ± 0.01	0.04 ± 0.01	0.04 ± 0.01
Liver FFA (μM)	3.15 ± 0.43	4.98 ± 0.21 *	4.17 ± 0.36 *^#^
Liver β-HB (mM)	0.17 ± 0.01	0.15 ± 0.01 *	0.16 ± 0.01 ^#^
Serum ALT (U/L)	14.00 ± 3.98	83.11 ± 12.63 *	21.76 ± 9.41 ^#^
Serum AST (U/L)	17.16 ± 5.18	32.07 ± 4.68 *	23.09 ± 5.09 ^#^
Liver TNF-α (ng/mL)	0.33 ± 0.03	0.49 ± 0.07 *	0.32 ± 0.04 ^#^
Liver IL-6 (ng/mL)	0.06 ± 0.01	0.10 ± 0.02 *	0.03 ± 0.02 ^#^

All values are presented as mean ± SD (*n* = 5). * *p* < 0.05 versus the NFD group; ^#^ *p* < 0.05 versus the HFD group.
